# Fluorine polymer probes for magnetic resonance imaging: quo vadis?

**DOI:** 10.1007/s10334-018-0724-6

**Published:** 2018-11-29

**Authors:** Daniel Jirak, Andrea Galisova, Kristyna Kolouchova, David Babuka, Martin Hruby

**Affiliations:** 1 Institute for Clinical and Experimental Medicine, Vídeňská 9, 140 21 Prague 4, Czech Republic; 2 Institute of Biophysics and Informatics, 1st Medicine Faculty, Charles University, Salmovská 1, 120 00 Prague, Czech Republic; 3 Faculty of Health Studies, Technical University of Liberec, Studentská 1402/2, 461 17 Liberec 1, Czech Republic; 4 Institute of Macromolecular Chemistry, Czech Academy of Sciences, Heyrovského sq. 2, 162 06 Prague 6, Czech Republic

**Keywords:** Fluorine, Magnetic resonance imaging (MRI), Polymer, ^19^F MRI probe, Molecular imaging

## Abstract

Over the last few years, the development and relevance of ^19^F magnetic resonance imaging (MRI) for use in clinical practice has emerged. MRI using fluorinated probes enables the achievement of a specific signal with high contrast in MRI images. However, to ensure sufficient sensitivity of ^19^F MRI, fluorine probes with a high content of chemically equivalent fluorine atoms are required. The majority of ^19^F MRI agents are perfluorocarbon emulsions, which have a broad range of applications in molecular imaging, although the content of fluorine atoms in these molecules is limited. In this review, we focus mainly on polymer probes that allow higher fluorine content and represent versatile platforms with properties tailorable to a plethora of biomedical in vivo applications. We discuss the chemical development, up to the first imaging applications, of these promising fluorine probes, including injectable polymers that form depots that are intended for possible use in cancer therapy.

## Introduction

Magnetic resonance imaging (MRI) using fluorinated probes (^19^F MRI) enables high contrast in images due to the negligible fluorine background of living tissues. For this reason, fluorine isotopes are visualized with high specificity, allowing for so-called “hot spot” imaging where the ^19^F MR signal is overlaid on a proton reference MR image. Moreover, the fluorine isotope ^19^F resonates at a Larmor frequency close to that of the proton ^1^H (94% of ^1^H); therefore, the hardware used for ^1^H MR imaging, with minor modifications, can also be used for ^19^F MRI. Currently, many clinical and experimental scanners have become available for hardware modifications and the use of appropriate radiofrequency (RF) coils. Usually, the RF coils intended for ^19^F MR are constructed to be dual-tuned for both ^1^H/^19^F MRI. The ^19^F MR signal is proportional to the number of ^19^F nuclei, which allows absolute quantification of the ^19^F content.

For ^19^F MRI, fluorine-containing probes need to be synthesized and then administered into the organism by either injection (intravenously or locally to the site of interest) or exogenous labeling of cells. Currently, there is a wide range of available fluorine probes for experimental and even clinical applications summarized in many excellent reviews [1–4]. The implementation of ^19^F MR probes ranges from cell targeting [5–8] and in vivo tracking of labeled cells [9–18], cancer diagnosis [19–22], inflammation monitoring [23–26], amyloid plaque detection or suppression [27–29], in situ partial oxygen pressure (pO_2_) determination [30, 31], drug metabolism (e.g., of 5-fluorouracil) investigation [32], intra/extracellular pH measurement [33, 34] or cation concentration estimation in cells and tissues [35]. In humans, the ^19^F probes have been tested in the immunotherapy of colorectal cancer with labeled dendritic cells, and a number of other clinical trials are ongoing [3, 12]. Currently, several theranostic ^19^F MR probes combining both diagnosis and therapy [36] have been implemented; however, testing of the probes on biological models of diseases is far behind their constantly evolving chemical development.

The main drawback of ^19^F MR imaging is its low sensitivity due to the low amount of ^19^F nuclei per molecule of the synthesized fluorine-based probes. Usually, the concentration of ^19^F atoms in fluorine-based probes is in the millimolar range (for comparison, water has a proton concentration of approximately 110 M under ambient conditions); therefore, agents containing a large number of ^19^F atoms are necessary for a sufficiently high signal-to-noise ratio (SNR). For a well-resolved single spectral peak, the key property needed for fluorine probes is an as-high-as-possible fraction of chemically equivalent ^19^F atoms in the molecule. A single peak allows us to detect a higher ^19^F MR signal compared to multiple spectral peaks, which can cause chemical shift artifacts in the MR images.

These demands can be accomplished, e.g., by probes based on polymer structures. Polymer probes enable the incorporation of a large amount of chemically equivalent fluorine atoms into a single molecule and thus could overcome the sensitivity issue of ^19^F MRI. Moreover, polymer probes can be easily modified to increase their biological response and improve their behavior in living systems (pH, thermoresponsive parts or bonds, which might be enzymatically degraded) or to add the imaging labels to drug moieties. In this review, we summarize the current state of the art for polymer-based ^19^F MRI probes that have great potential in human medicine.

## Types of fluorine-containing probes

Various types of fluorinated agents are available in the form of fluorinated perfluorocarbon (PFC) nanoemulsions [37], fluorinated lanthanide chelates [2, 6], fluorinated nucleotides [32], fluoride-based nanocrystals [38], multicompartment amphiphilic polymers [19, 39–41], etc. A brief overview of fluorine-containing probes, including their systematic names, abbreviations, chemical structures, molecular weights and fluorine content, can be found in Table 1.

**Table 1:**
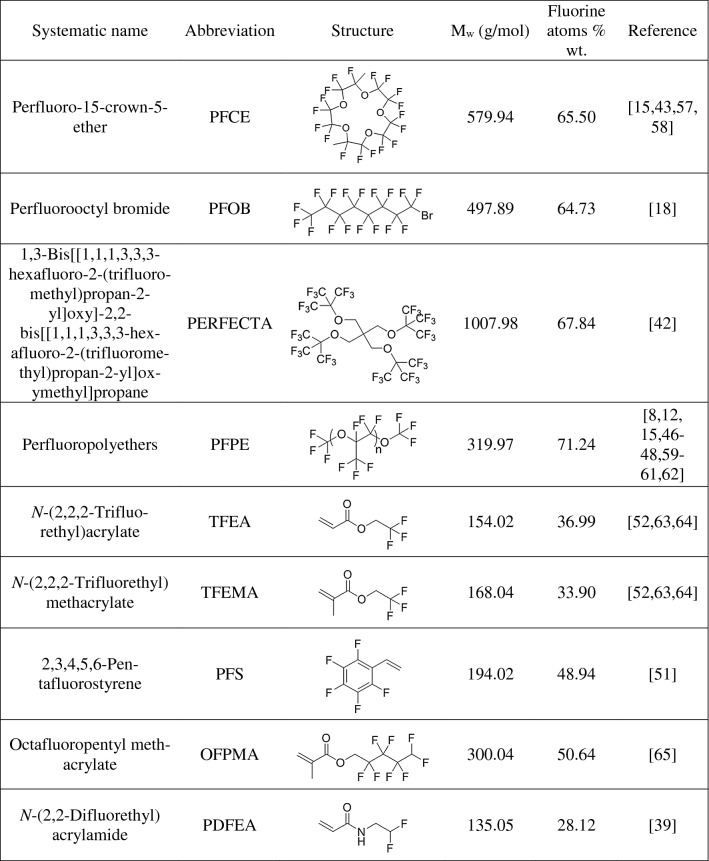
Overview of the most commonly used fluorine-containing probes

The most frequently used ^19^F MR agents are liquid PFCs, e.g., perfluoro-15-crown-5-ether (PFCE) (containing 20 fluorine atoms), perfluorooctyl bromide (PFOB) (17 fluorine atoms), a superfluorinated probe called PERFECTA (bearing 36 chemically equivalent fluorine atoms per molecule) [42], or perfluoropolyethers (PFPE) (with more than 40 fluorine atoms per molecule). These probes possess convenient features for in vivo applications such as nontoxicity, biological and chemical inertness and the ability to be internalized into cells, enabling their labeling. In this way, e.g., macrophages, stem cells, immune cells [8, 11, 13], or even clusters of cells such as pancreatic islets [17, 43] may be visualized and tracked. PFCs are both hydrophobic and lipophobic, and thus, liquid PFC emulsions must be stabilized with surfactants (lipids or phospholipids) or entrapped in polymer nanoparticles [44, 45] for stabilization and biocompatibility [15, 44]. There are many preclinical applications using PFC-based agents covering the imaging of cardiac progenitor stem cells and bone-derived bone marrow macrophages [15], detection of dendritic cells [46], visualization of the immune response [47, 48], tracking of cells or pancreatic islets [43], etc. PFCs with a single ^19^F MR peak in their spectra have been implemented in various studies, including a clinical trial with labeled dendritic cells (DCs) intended for adenocarcinoma treatment [12]. Although widely implemented, PFCs also have limitations, such as nonspecific accumulation in tissues or at the site of inflammation [49], possible macrophage activation after high dosage [50], altered cellular responses due to the incorporation of PFCs into the cellular membranes [1] and long retention times in the body.

In addition to cell labeling and tracking, fluorine-containing probes can also be implemented for other purposes. Recently, polymer agents consisting of molecular hydrophobic and hydrophilic blocks (copolymers) have shown promising properties for biomedical applications, especially for tumor diagnosis based upon their responsiveness to the chemical environment or as possible drug carriers due to their nanoscale size. These probes can carry large amounts of fluorine atoms, resulting in a high sensitivity of visualization, although their short relaxation times resulting from polymerization need to be adjusted. In the majority of these amphiphilic agents, the fluorinated block is based on acrylic acid, specifically on 2,2,2-trifluorethylacrylate (TFEA), 2,2,2-trifluorethyl methacrylate (TFEMA), poly[*N*-(2,2-difluorethyl)acrylamide] (PDFEA) [39, 40] or octafluoropentyl methacrylate (OFPMA). There are also polymers based on functionalized styrenes (e.g., 2,3,4,5,6-pentafluorostyrene (PFS)) [51] or polydimethylsiloxanes (PDMS) [52]. These probes are described in more detail in the section “Polymer agents for drug delivery”

Fluorine-containing agents can also be targeted to specific structures (e.g., the cell surface); accumulated in specific cells such as macrophages, stem cells and immune cells [1, 53]; and responsive to specific stimuli in the environment (physiological processes, enzymatic activity, concentrations of metal ions, pH or pO_2_) [3, 35, 54–56].

## Preparation of fluorinated probes

The most commonly employed probes are PFCs, which are typically used as stabilized aqueous (nano)emulsions prepared under high pressure; emulsification is usually performed by probe sonication. An emerging class of ^19^F probes are fluorinated polymers. These polymers can be used either alone or as colloidal stabilizers for PFCs. Fluorinated copolymers are typically synthesized analogously to their nonfluorinated counterparts, i.e., by (controlled pseudo living) radical, cationic, anionic or coordination polymerization. The most commonly used controlled radical polymerization techniques involve nitroxide-mediated polymerization [66, 67], atom transfer polymerization (ATRP) [68, 69] and reversible addition fragmentation chain transfer (RAFT) polymerization [70, 71]. It is worth mentioning that if multiple fluorine atoms are in close proximity to the polymerizable moiety, then they may dramatically reduce the electron density of the moiety, greatly influencing its reactivity to polymerization. Other procedures to introduce fluorine atoms into polymer structures involve fluoroalkanoyl peroxide initiation [72, 73], telomerizations with fluorinated mercaptan and fluorinated iodides [74] and esterification with perfluoroacyl chloride [75]. The fluorine block can also be introduced within the termination process [76]. The various copolymer structures include linear copolymers, hyperbranched copolymers, dendrimers [77–79], micelles [80] and star- and knedel-like structures [51, 65].

## Properties of fluorinated polymers

A high density of equivalent fluorine atoms is required in the probes suitable for ^19^F MRI. However, (per)fluorinated—fluorophilic parts of the molecules/polymer blocks are naturally insoluble in water and aggregation of ^19^F probes with high fluorine content is often observed in aqueous solution. This causes attenuation of MR signal and decreasing the ^19^F MR sensitivity. Therefore, it is necessary to solubilize them in aqueous milieus for their use as contrast agents. This may be done by adding covalently bound sufficiently hydrophilic moieties or polymer block(s) or by noncovalent incorporation of fluorophilic molecules into supramolecular assemblies containing amphiphiles solubilizing the whole system in aqueous milieu.

The specific advantage of polymer systems, especially from a diagnostic and therapeutic point of view, is the possibility to construct delivery systems for drugs, nucleic acids, imaging labels or radionuclides to target cancerous tissue, macrophages or other cells and their subcellular compartments. The polymer systems can easily build “molecular toolboxes”, which can be targeted and used as efficient theranostic tools [81]. Self-assembled (supramolecular) polymer systems are very promising. Supramolecular polymer systems are assembled from many molecules that can be bound together by various noncovalent interactions, such as hydrophobic, ion–ion, and coordination interactions [82, 83]. The degradability of such systems may be controlled not only chemically but also physically. According to the type of bonds and molecules, different polymer systems can be created, such as polymer micelles, polymersomes, polyplexes, and polymer nanoparticles [76, 84]. Many fluorinated polymers exhibit self-assembly properties, which are based on the ability of fluorinated molecules to phase separate and assemble into a fluorous phase that is both hydrophobic and lipophobic. The advantage of the self-assembly feature is its dependency on environmental conditions; therefore, these agents can act primary as environmentally responsive probes. These systems are responsive to external stimuli, such as changes in pH, temperature, redox potential, enzymes, and ion concentrations. Their features can be used for custom-built self-assembling scaffolds and depots.

From an imaging point of view, the challenge in synthesizing multiblock copolymers is ensuring high fluorine contents and suitable relaxation times for ^19^F MRI. Under optimal conditions, the *T*_1_ relaxation time should not be too long for recovery of the longitudinal magnetization; *T*_1_ relaxation times of many fluorinated molecules are in the range of 1–4 s, which results in long acquisition times for in vivo experiments [85, 86]. Fluorine *T*_2_ relaxation times are based on the mobility of the fluorine segments and need to be adjusted to permit imaging by standard spin, gradient and ultrashort echo time (UTE) sequences [37], which means the *T*_2_ relaxation times should not be too short (> 10 ms). Adjustment of the relaxation times is crucial and challenging, and various chemical approaches have been tested [53, 87–89]. For instance, control over the molecular mobility can be achieved by preventing the very strong fluorine–fluorine interactions by hydration of the fluorosegments [19]. Moreover, the association of the fluorine segments needs to be taken into account due to strong dipole–dipole interactions and shortening of the relaxation times [63]. The proximity of paramagnetic atoms, such as lanthanides, also highly influences fluorine relaxation.

From a diagnostic point of view, fluorine-containing contrast agents possess the added value of specificity and responsiveness. The specificity can be achieved by modification with a targeting moiety; therefore, it is crucial to prepare easily modifiable chemical structures for the probes.

In summary, a polymer probe suitable for ^19^F MRI should possess:

1. a high content of equivalent fluorine atoms so that all of them fit into the chemical shift window of interest,
2. efficient mobility and limited association resulting in suitable relaxation times,
3. adequate solubility in water,
4. an easily modifiable structure for targeting, and
5. reliable pharmacokinetic and pharmacodynamic properties, including biodegradability and elimination after the probe fulfills its task

### Polymer perfluorocarbons

The commercially available PFC-based probes include the PFPE-based agents Cell Sense and V-Sense (CS-1000-DM-Red and VS-1000H, Celsense, Inc., Pittsburgh, USA). These types of probes were introduced in the 1980s for tumor detection [60, 61], and their use as a cell label was shown for the first time in 2005 [8]. Although PFPE-based agents have been implemented in various preclinical and clinical studies, they are difficult to further functionalize, and the possible extra covalent bond would break the symmetry of the probe, which could lead to multiple ^19^F peaks [42]. Therefore, there is a need for the synthesis of novel probes with higher fluorine content and the potential for easy modification. In 2017, Zhang et al. [62] synthesized a polymer agent based on PFPE with a high fluorine content between 10 and 29% wt. The polymer consists of PFPE end-functionalized homopolymers of oligo(ethylene glycol) methyl ether acrylate (poly(OEGA)m-PFPE). These polymers are water-soluble and possess outstanding imaging sensitivity as was shown with in vitro and in vivo experiments. The long *T*_2_ relaxation times (> 80 ms) of these PFPE-based probes are suitable for imaging using common MR sequences, such as a spin-echo-based sequence. The polymers accumulated in the liver, kidneys and spleen of mice after intravenous administration of the probe. The difference in the ratio of hydrophobic (PFPE) and hydrophilic [oligo(ethylene oxide) methyl ether acrylate] segments played an important role in the accumulation of the probe in biological tissue. The polymer showed resistance to rapid uptake by macrophages and thus longer circulation times in the blood. This effect is also seen in other studies [90, 91]. The polymer with exposed PFPE segments showed enhanced recognition and filtration by macrophages, resulting in faster clearance from the body.

### Fluorine molecular blocks

#### Development of fluorine copolymers for ^19^F MRI: from chemical concept to first in vivo ^19^F MR images

Since the first reports on the synthesis of copolymers consisting of fluorinated molecular blocks, these probes have been extensively studied. In 2007, Cheng et al. [52] introduced a hyperbranched fluorinated copolymer consisting of a fluorinated backbone and PDMS. The proposed cross-linking of the hyperbranched fluoropolymers with PDMS or poly(ethylene oxide) (PEO) showed unique properties, such as an anti-biofouling ability, release behavior for various molecules and mechanical performance [92]. This approach inspired the further synthesis of ^19^F copolymers intended for ^19^F MRI. In 2008, the Wooley group synthesized polymers of trifluorethyl methacrylates and acrylic acid grafted onto a hydrophobic hyperbranched core. The fluoropolymers possessed good imaging properties (*T*_1_/*T*_2_ = 500/50 ms) and achieved a high SNR in a phantom study, although with an extensive scanning time (13 h) [93]. Later, this group studied the imaging performance of styrene-based polymers and observed that the packaging of fluorine-rich segments into the core restricts the mobility of the chains and limits ^19^F detectability [51]. The polymers had to be dissolved in dimethyl sulfoxide (DMSO) to increase their mobility, which increased the sensitivity of ^19^F MR signal detection in MR spectra; therefore, this approach is not suitable for biological applications due to the toxicity of DMSO.

The first report of ^19^F MRI of fluorinated copolymers was published in 2009 by Peng et al. [64]. This group synthesized diblock copolymers of acrylic acid with partially fluorinated acrylate or methacrylate monomers, which undergo spontaneous assembly in mixed or aqueous solvents resulting in the formation of micelles (diameter 20-45 nm). The micelles exhibited a strong signal in ^19^F MR images, with a higher signal obtained from the methacrylate polymers compared to the acrylate polymers due to the short *T*_2_ relaxation times of the acrylate. The ^19^F *T*_1_ relaxation times were approximately 500 – 600 ms, and the ^19^F *T*_2_ relaxation times were 331 ms and 249 ms for TFEA and TFEMA, respectively. These ranges for the *T*_1_ and *T*_2_ relaxation times are suitable for visualization with routinely used MR imaging sequences without the need to use UTE or zero echo time (ZTE) sequences. The ^19^F MR images of phantoms containing the copolymers were acquired within 1 h 20 min using a 3D spin-echo sequence at 7 T (2.5 mm slice thickness). The low ^19^F MR signal detection attributed to the restricted mobility of the fluorinated segments; however, the group later introduced various approaches to increase the mobility of the fluorinated segments while maintaining the high amount of ^19^F atoms in a molecule. In 2010, the group reported an approach to enhance the hydration of the fluorinated blocks by preventing their aggregation in water by exploiting the electrostatic repulsion between monomeric units, which contributed to higher detectability [94].

Then, in 2010, highly flexible hyperbranched polymers that maintained the molecular mobility of the fluorinated chains were introduced by Thurecht et al. [80]. Moreover, this cytocompatible hyperbranched polymer can be easily functionalized for tracking or targeting. The polymer possesses “shape-resistance”, which is important for cell targeting and ensures the proper orientation for biological recognition. To eliminate the toxic effect of the cationic hyperbranched core, PEO monomethylether methacrylate (PEOMA) was incorporated into the molecule. Importantly, the polymer had been modified with mannose for biological targeting, e.g., the immune responses mediated by macrophages, which possess DC-SIGN surface receptors for mannosylated species. Fluorine *T*_2_ relaxation times of the polymers were in the detectable range (68–122 ms).

The same group reported a study focused on testing the solvent influence on an assembly of TFEA/TFEMA-based polymers [63]. Diffuse aggregates were formed in dichloromethane and micelles were formed in acetone. After the addition of water to these systems, both systems formed cylindrical structures. The highest ^19^F MR signal was observed from polymers dissolved in acetone, which highlights the dependence of imaging properties on the solvent used due to the rigidity of the structure. Association of the fluorine segments leads to strong dipole–dipole interactions between the ^19^F spins, causing shortening of the ^19^F spin–spin relaxation times and therefore lowering the ^19^F MR detectability. To overcome the effect of dipole–dipole interactions, several approaches for improving MRI detectability were proposed, such as distributing the monomers along the polymer chain, incorporating branches and limiting the content of the fluorinated monomers [63]. Although this study was performed in biologically irrelevant organic solvents, it reveals rules of general applicability.

#### Fluorine copolymers responsive to environmental changes: from optimization of the chemical structure to biological applications

In addition to the use of fluorine copolymers for contrast modulation in ^19^F MR images, various probes also respond to environmental stimuli and can act as responsive probes for, e.g., controlled in situ supramolecular nanostructure formation due to external environment change or even responsive ^19^F MRI. The rationale for such a responsive agent would be, e.g., the evaluation of treatment response by changes in pH, controlled release of drugs upon changes in pH, and accumulation of the system at the target site according to pH (acidity in tumors).

The agents mostly respond to changes in pH or temperature by triggering self-assembly. For the synthesis and application of these types of polymers, it is crucial to achieve a lower critical solution temperature (LCST) within an adequate range. The LCST refers to the temperature below which the components of the polymer are miscible. At temperatures above the LCST, polymers lose their hydration layer, start to precipitate and change into a solid phase. As a macroscopic measure, the value most often used is the cloud point temperature (CPT), which is where the polymer visibly phase-separates at the given concentration, where LCST is the temperature minimum in the function CPT = f(concentration). As the components become less mobile, the relaxation times are shortened. These aggregation properties can be implemented in the creation of solid implants without the need for surgical intervention [40, 95].

In 2007, the group of Mao et al. [65] presented amphiphilic hyperbranched star-block copolymers containing poly[2-(*N*,*N*-dimethylamino)ethyl methacrylate] (PDMAEMA) and OFPMA, which self-assemble into micelles in either an acidic aqueous solution or in a dimethylformamide/water mixture (both at pH 3). The size (expressed as hydrodynamic diameter, *D*_H_) of the micelles depends on the length of the DMAEMA segment and varies with pH. The authors reported various sizes (50–200 nm) and structures for the synthesized probes ranging from micelles to fibers depending on the length of the protonated PDMAEMA chains. Fluorine MR spectra showed multiple ^19^F peaks for the polymer.

Similar thermo- and pH-responsive fluoroalkyl end-capped amphiphilic diblock copolymers containing PDMAEMA and poly[2-(*N*,*N*-diethylamino)ethyl methacrylate] (PDEA) were synthesized by the Zhang [96]. These polymers can form flower-like micelles in aqueous solution upon pH changes or by changing the linking order of the PDMA and PDEA blocks. The LCST of these polymers decreases with increasing concentration or pH. At a low pH of approximately 3, there is no aggregation, and the copolymers exist in the aqueous solution as unimers. At a higher pH (7- 9), the copolymer forms micelles, and at a pH of approximately 9, the immiscible components aggregate into a core. By changing the linking order of the PDMA and PDEA blocks and changing the pH, different morphologies were obtained such as sphere-on-sphere, flower-like micelles and anomalous vesicles. Fluorine MR spectroscopy showed three ^19^F peaks in the spectra of the polymers.

In another study, PEO-coated nanogels constructed from PEO chains and a polyamine gel core compound with ^19^F showed suitable properties for targeting solid tumors via their responsiveness to the low pH (6.5) present in the tumor environment [20]. The polymer prepared from 2-(*N*,*N*-diethylamino)ethyl methacrylate (DEAMA) and TFEMA at various molar ratios with PEO chains showed volume phase transition points in the pH range of 6.8–7.3. This effect is caused by the hydrophobicity of the gel core and deprotonization of the amino groups, leading to a broadening of the ^19^F MR signals due to the limited molecular motion, suggesting that the PEO-coated nanogels will swell only in the acidic environment of, e.g., tumors (pH 6.5–7.0). This effect was confirmed by ^19^F MR spectroscopy; the polymers were only detected at acidic pH, and there was almost no ^19^F MR signal in the physiological pH range (7.4). A stable on–off behavior was observed even in the presence of 90% fetal bovine serum, which is promising for future biological applications. The probe showed high sensitivity (the detection threshold as measured by ^19^F MR spectroscopy was 55 µM at 12 T), although ^19^F MR imaging was not performed in this study.

## Polymer agents for drug delivery

In theranostics (therapy + diagnosis), nanoparticles and micelles (with *D*_H_, below 200 nm) can be used for passive targeting of solid tumors due to the enhanced permeability and retention effect (EPR) [97–99], which is caused by the leaky vasculature and limited lymphatic drainage of solid tumor tissue. Moreover, the probes can be further functionalized to prolong their circulation times [e.g., with PEO or poly(2-methyl-2-oxazolines)] and thus increase their probability of accumulation in tumor tissue [100–103].

The first example of ^19^F MR imaging of the accumulation of fluoropolymers in tumors was reported in 2014 by Rolfe et al. 2014 [19]. In this study, a tunable polymer probe, which targeted melanoma cells and enabled multimodal imaging due to the addition of a fluorescent dye, was introduced. The probe was further functionalized for tumor targeting via modification with folate. The probe was found to be taken up by B16 melanoma cells, which overexpress folate receptors. The probe was detected in the tumors of mice after intravenous administration. Fluorescence and ^19^F MR images revealed the accumulation of the probe in the major organs (the liver, kidneys and bladder) 4 h following injection. ^19^F MR images of the mice were acquired within 18 min at 16.4 T.

In 2017, the nanoparticles of block copolymers containing a PEO hydrophilic block and a fluorine-containing polymethacrylate block were synthesized by Fu et al. [104]. These polymer nanoparticles exhibit a higher ^19^F MR signal in the presence of H_2_O_2_ (i.e., after micelle disassembly due to hydrophilization after oxidation of the thioethers to sulfoxide), suggesting a promising direction for future biological applications. The sensitivity of the imaging agents was further enhanced by adding a pH switch, resulting in a reactive oxygen species (ROS)/pH dual-responsive ^19^F MRI agent. The most pronounced change in the intensity of the ^19^F MR signal was achieved in response to the presence of ROS in a mildly acidic environment.

**Fig. 1:**
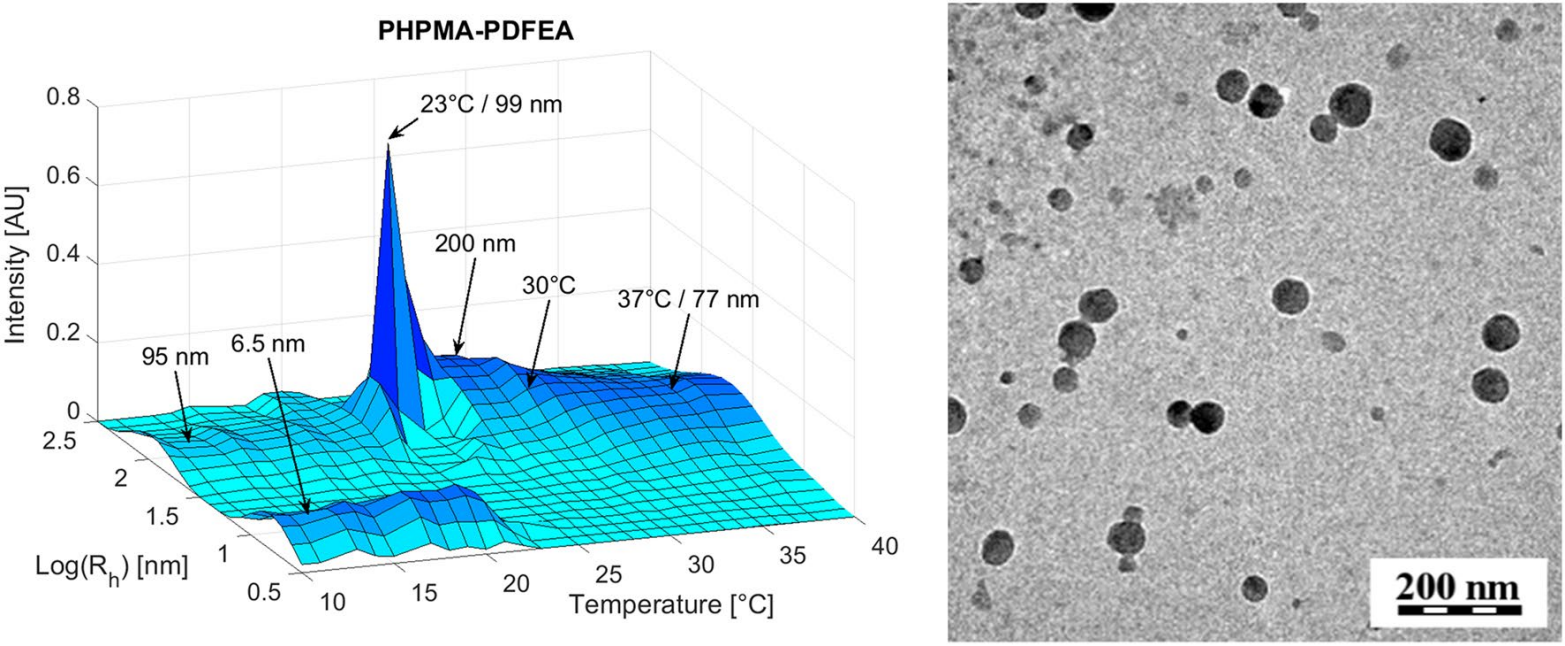
Thermal self-assembly of a PHPMA-PDFEA copolymer in phosphate-buffered saline (PBS) buffer at temperatures above 23 °C: 3D graph of the dependence of particle size distribution as measured by dynamic light scattering (DLS) on temperature (left) and transmission electron cryomicroscopy (CryoTEM) image of the formed nanoparticles (right). The polymer remains molecularly dissolved (monomers—*R*_h_ approx. 6.5 nm) at temperatures below 23 °C and undergoes self-assembly into nanogel particles (hydrodynamic radius R_h_ approx. 77 nm at 37 °C) above this temperature [39]

**Fig. 2:**
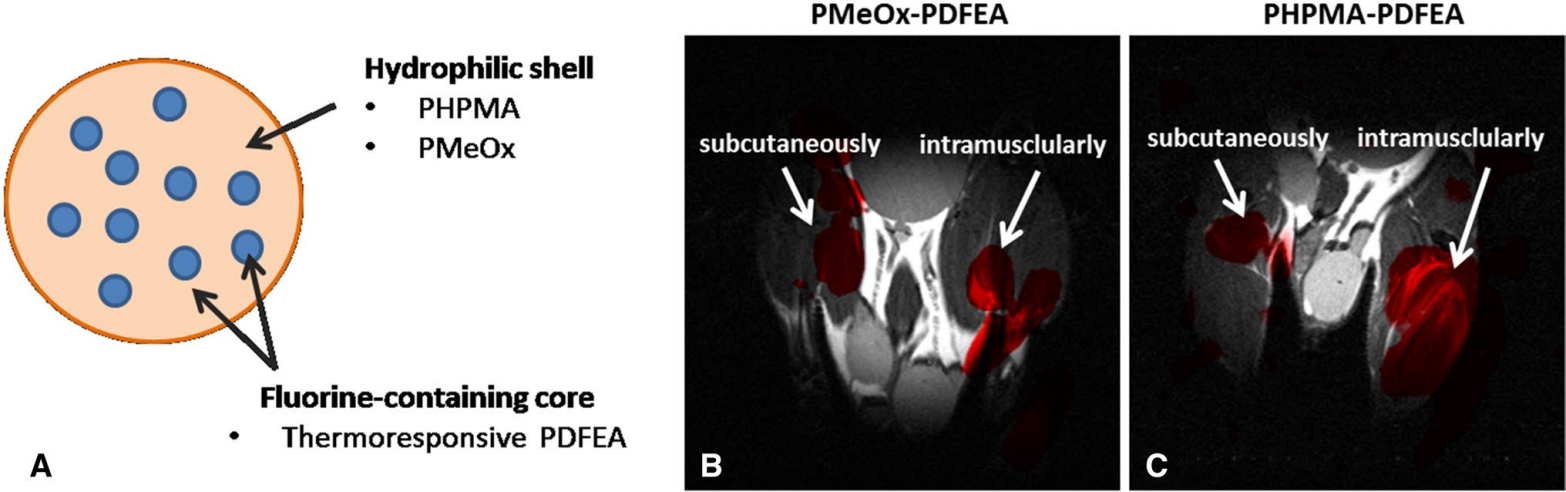
A scheme of self-assembling nanoparticles containing a hydrophilic shell and a fluorine-containing core (**a**). ^19^F MR images (red color) superimposed on ^1^H (grayscale) MR images of mice injected with probes: PMeOx-PDFEA (**b**) and PHPMA-PDFEA (**c**). The acquisition time of the ^19^F MR images was 17 min at 4.7 T. The arrows indicate the injection sites

Self-assembled biocompatible polymer nanogels consisting of hydrophilic-thermoresponsive diblock copolymers containing either poly[*N*-(2-hydroxypropyl)methacrylamide] (PHPMA) or poly(2-methyl-2-oxazoline) (PMeOx) as the hydrophilic block and PDFEA as the fluorinated thermoresponsive block exhibited thermal responsivity, being molecularly soluble at room temperature and forming well-defined nanogels at body temperature (Fig. 1) [39]. The probes exhibit suitable relaxation times (*T*_1_ ≈ 275–312 ms, *T*_2_ ≈ 17–305 ms; at 4.7 T, 37 °C) for visualization by ^19^F MRI using standard spin-echo sequences. The presence of both polymers (PMeOx-PDFEA and PHPMA-PDFEA) was observed by ^19^F MRI after injection either into the muscle of mice or subcutaneously (Fig. 2). These probes have promising properties for cell tracking or tumor theranostics due to their easily modifiable structure, for incorporation of cell targeting moieties, and their nanosize (approximately 100 nm), allowing passive accumulation in the tumor tissue via the EPR effect.

**Fig. 3:**
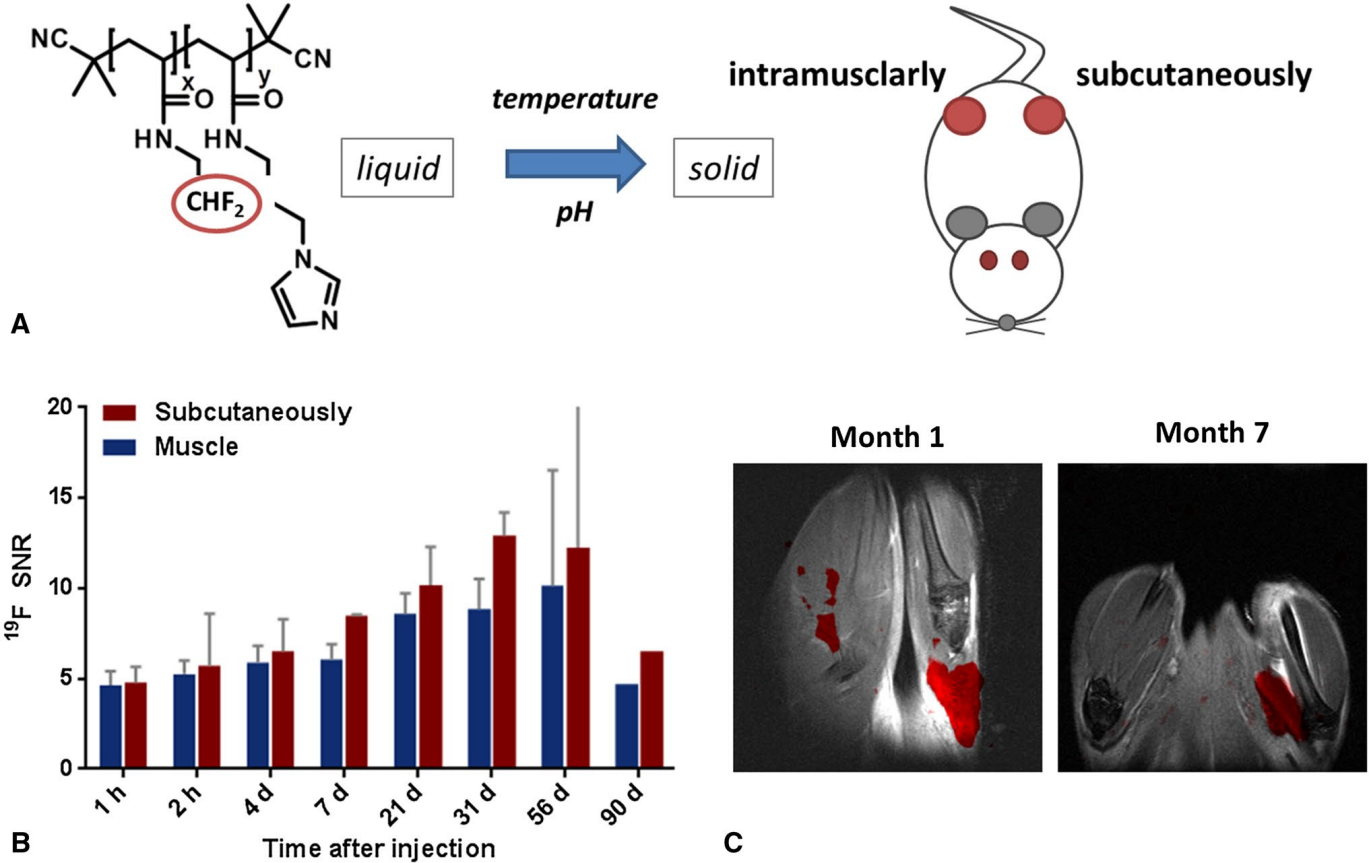
The pH/thermoresponsive polymers change phase from liquid to solid after injection into the body (intramuscularly and subcutaneously in mice) (**a**). Quantification of the ^19^F MR signal by the SNR at the different injection sites (**b**) and ^19^F MR images confirming the creation of polymer depots at the injection sites, resulting in a strong ^19^F MR signal in the long term (**c**)

Fluorinated thermoresponsive polymers can also be implemented for sustained release of drugs from implants, which are created upon injection into the body [105]. Typically, the polymers are injected in an aqueous solution, and after heating to body temperature, which is above the CPT, they form a depot [95, 106]. Above the LCST, the polymers precipitate, and after phase separation, they create a solid depot without surgery [95]. Moreover, if the polymers contain ^19^F atoms, the localization, size and biodegradation of the implants can be monitored by ^19^F MRI with high sensitivity due to the high local ^19^F concentration at the injection site. In our recent study [40], we used a thermoresponsive fluorinated PDFEA copolymer modified with pH-responsive imidazole units, where the pH change from slightly acidic in the injected solution to neutral in the tissue adjusts CPT, allowing for in situ depot formation due to temperature change without the risk of needle obstruction during injection. This multistimuli-responsive agent was tracked by ^19^F MRI after intramuscular and subcutaneous administration in rats (Fig. 3). The depot was visualized for 11 months with high sensitivity, indicating that these thermoresponsive polymers can be utilized as injectable solid implants with the possibility of drug incorporation. Importantly, the degradability of the polymers can be tailored in future theranostic applications.

## Conclusion and future perspectives

^19^F MRI using fluorine-containing agents represents a specific imaging method with applications in both experimental and clinical medicine due to the negligible ^19^F MR signals from the body. To achieve sufficient sensitivity of ^19^F MRI, novel fluorine-containing probes are needed. Polymer-based probes are a very promising and versatile platform with properties tailorable to a plethora of in vivo biomedical applications. In particular, stimuli-responsive supramolecular polymer nanostructures allowing for functional imaging in response to changes in temperature, pH, reactive oxygen species and other physiological stimuli hold promise for noninvasive functional diagnostics of pathological tissues in the future. Targeted cell labeling for long-term tracking of transplanted cells by ^19^F MRI is another highly promising direction for such ^19^F agents.

To employ the ^19^F MRI polymer probes in practice, especially in clinics, several properties of these probes must be fulfilled. Among them are a high content of fluorine atoms that are chemically equivalent so that all of them fit into the chemical shift window of interest, an efficient mobility and limited association of the fluorine segments resulting in suitable relaxation times, an adequate solubility in water, an easily modifiable structure for targeting, reliable pharmacokinetic/pharmacodynamic properties including biodegradability, clearance of the probe after the system fulfills its task, and the capability of functional imaging if needed in the particular application. Another important challenge that must be solved is the biodegradability of the imaging agents so that the system may be fully eliminated from the organism after fulfilling its task.

## Abbreviations

ATRP: Atom transfer polymerization
CPT: Cloud point temperature
CryoTEM: Transmission electron cryomicroscopy
DCs: Dendritic cells
DEAMA: 2-(*N*,*N*-Diethylamino)ethyl methacrylate
DLS: Dynamic light scattering
DMSO: Dimethyl sulfoxide
EPR: Enhanced permeability and retention effect
LCST: Lower critical solution temperature
MRI: Magnetic resonance imaging
OFPMA: Octafluoropentyl methacrylate
PBS: Phosphate-buffered saline
PDEA: Poly[2-(*N*,*N*-diethylamino)ethyl methacrylate]
PDFEA: Poly[*N*-(2,2-difluorethyl)acrylamide]
PDMAEMA: Poly[2-(*N*,*N*-dimethylamino)ethyl methacrylate]
PDMS: Polydimethylsiloxanes
PEO: Poly(ethylene oxide)
PEOMA: PEO monomethylether methacrylate
PERFECTA: Superfluorinated probe
PFC: Perfluorocarbon
PFCE: Perfluoro-15-crown-5-ether
PFS: 2,3,4,5,6-Pentafluorostyrene
PFOB: Perfluorooctyl bromide
poly(OEGA)m-PFPE: Oligo(ethylene glycol) methyl ether acrylate
PFPE: Perfluoropolyethers
PHPMA: Poly[*N*-(2-hydroxypropyl)methacrylamide]
PMeOx: Poly(2-methyl-2-oxazoline)
RAFT: Reversible addition fragmentation chain transfer
RF: Radiofrequency
ROS: Reactive oxygen species
SNR: Signal-to-noise ratio
TFEA: 2,2,2-Trifluorethylacrylate
TFEMA: 2,2,2-Trifluorethyl methacrylate
UTE: Ultrashort echo time
ZTE: Zero echo time

## References

[1] Ruiz-Cabello J, Barnett BP, Bottomley PA, Bulte JWM (2011). Fluorine (F-19) MRS and MRI in biomedicine. NMR Biomed.

[2] Peterson KL, Srivastava K, Pierre VC (2018) Fluorinated paramagnetic complexes: sensitive and responsive probes for magnetic resonance spectroscopy and imaging. Front Chem 6 10.3389/fchem.2018.00160 PMC5974164 29876342

[3] Yu JX, Hallac RR, Chiguru S, Mason RP (2013). New frontiers and developing applications in F-19 NMR. Prog Nucl Mag Res Sp.

[4] Srinivas M, Heerschap A, Ahrens ET, Figdor CG, de Vries IJM (2010). F-19 MRI for quantitative in vivo cell tracking. Trends Biotechnol.

[5] Bulte JW (2005). Hot spot MRI emerges from the background. Nat Biotechnol.

[6] Blahut J, Bernasek K, Galisova A, Herynek V, Cisarova I, Kotek J, Lang J, Matejkova S, Hermann P (2017). Paramagnetic (19)F relaxation enhancement in Nickel(II) complexes of *N*-trifluoroethyl cyclam derivatives and cell labeling for (19)F MRI. Inorg Chem.

[7] Knight JC, Edwards PG, Paisey SJ (2011). Fluorinated contrast agents for magnetic resonance imaging; a review of recent developments. Rsc Adv.

[8] Ahrens ET, Flores R, Xu H, Morel PA (2005). In vivo imaging platform for tracking immunotherapeutic cells. Nat Biotechnol.

[9] Fink C, Gaudet JM, Fox MS, Bhatt S, Viswanathan S, Smith M, Chin J, Foster PJ, Dekaban GA (2018). (19)F-perfluorocarbon-labeled human peripheral blood mononuclear cells can be detected in vivo using clinical MRI parameters in a therapeutic cell setting. Sci Rep.

[10] Gaudet JM, Ribot EJ, Chen Y, Gilbert KM, Foster PJ (2015). Tracking the fate of stem cell implants with fluorine-19 MRI. PLoS One.

[11] Srinivas M, Morel PA, Ernst LA, Laidlaw DH, Ahrens ET (2007). Fluorine-19 MRI for visualization and quantification of cell migration in a diabetes model. Magn Reson Med.

[12] Ahrens ET, Helfer BM, O’Hanlon CF, Schirda C (2014). Clinical cell therapy imaging using a perfluorocarbon tracer and fluorine-19 MRI. Magn Reson Med.

[13] Boehm-Sturm P, Aswendt M, Minassian A, Michalk S, Mengler L, Adamczak J, Mezzanotte L, Lowik C, Hoehn M (2014). A multi-modality platform to image stem cell graft survival in the naive and stroke-damaged mouse brain. Biomaterials.

[14] Tennstaedt A, Mastropietro A, Nelles M, Beyrau A, Hoehn M (2015). In vivo fate imaging of intracerebral stem cell grafts in mouse brain. PLoS One.

[15] Constantinides C, Maguire M, McNeill E, Carnicer R, Swider E, Srinivas M, Carr CA, Schneider JE (2018). Fast, quantitative, murine cardiac 19F MRI/MRS of PFCE-labeled progenitor stem cells and macrophages. PLoS One.

[16] Temme S, Bonner F, Schrader J, Flogel U (2012). 19F magnetic resonance imaging of endogenous macrophages in inflammation. Wiley Interdiscip Rev Nanomed Nanobiotechnol.

[17] Liang S, Louchami K, Holvoet B, Verbeke R, Deroose CM, Manshian B, Soenen SJ, Lentacker I, Himmelreich U (2018). Tri-modal in vivo imaging of pancreatic islets transplanted subcutaneously in mice. Mol Imaging Biol.

[18] Barnett BP, Ruiz-Cabello J, Hota P, Ouwerkerk R, Shamblott MJ, Lauzon C, Walczak P, Gilson WD, Chacko VP, Kraitchman DL, Arepally A, Bulte JW (2011). Use of perfluorocarbon nanoparticles for non-invasive multimodal cell tracking of human pancreatic islets. Contrast Media Mol Imaging.

[19] Rolfe BE, Blakey I, Squires O, Peng H, Boase NRB, Alexander C, Parsons PG, Boyle GM, Whittaker AK, Thurecht KJ (2014). Multimodal polymer nanoparticles with combined F-19 magnetic resonance and optical detection for tunable, targeted, multimodal imaging in vivo. J Am Chem Soc.

[20] Oishi M, Sumitani S, Nagasaki Y (2007). On-off regulation of F-19 magnetic resonance signals based on pH-sensitive PEGylated nanogels for potential tumor-specific smart F-19 MRI probes. Bioconjugate Chem.

[21] Young SW, Enzmann DR, Long DM, Muller HH (1981). Perfluoroctylbromide contrast enhancement of malignant neoplasms: preliminary observations. AJR Am J Roentgenol.

[22] Zhang C, Moonshi SS, Wang W, Ta HT, Han Y, Han FY, Peng H, Kral P, Rolfe BE, Gooding JJ, Gaus K, Whittaker AK (2018). High F-content perfluoropolyether-based nanoparticles for targeted detection of breast cancer by (19)F magnetic resonance and optical imaging. ACS Nano.

[23] Flogel U, Ding Z, Hardung H, Jander S, Reichmann G, Jacoby C, Schubert R, Schrader J (2008). In vivo monitoring of inflammation after cardiac and cerebral ischemia by fluorine magnetic resonance imaging. Circulation.

[24] Ahrens ET, Young WB, Xu H, Pusateri LK (2011). Rapid quantification of inflammation in tissue samples using perfluorocarbon emulsion and fluorine-19 nuclear magnetic resonance. Biotechniques.

[25] Murugesan R, English S, Reijnders K, Yamada K, Cook JA, Mitchell JB, Subramanian S, Krishna MC (2002). Fluorine electron double resonance imaging for 19F MRI in low magnetic fields. Magn Reson Med.

[26] Waiczies H, Lepore S, Drechsler S, Qadri F, Purfurst B, Sydow K, Dathe M, Kuhne A, Lindel T, Hoffmann W, Pohlmann A, Niendorf T, Waiczies S (2013). Visualizing brain inflammation with a shingled-leg radio-frequency head probe for 19F/1H MRI. Sci Rep.

[27] Fox MS, Gaudet JM, Foster PJ (2015). Fluorine-19 MRI contrast agents for cell tracking and lung imaging. Magn Reson Insights.

[28] Morawski AM, Winter PM, Yu X, Fuhrhop RW, Scott MJ, Hockett F, Robertson JD, Gaffney PJ, Lanza GM, Wickline SA (2004). Quantitative “magnetic resonance immunohistochemistry” with ligand-targeted (19)F nanoparticles. Magn Reson Med.

[29] Higuchi M, Iwata N, Matsuba Y, Sato K, Sasamoto K, Saido TC (2005). 19F and 1H MRI detection of amyloid beta plaques in vivo. Nat Neurosci.

[30] Winter PM, Morawski AM, Caruthers SD, Fuhrhop RW, Zhang H, Williams TA, Allen JS, Lacy EK, Robertson JD, Lanza GM, Wickline SA (2003). Molecular imaging of angiogenesis in early-stage atherosclerosis with alpha(v)beta3-integrin-targeted nanoparticles. Circulation.

[31] Thomas SR, Pratt RG, Millard RW, Samaratunga RC, Shiferaw Y, McGoron AJ, Tan KK (1996). In vivo PO2 imaging in the porcine model with perfluorocarbon F-19 NMR at low field. Magn Reson Imaging.

[32] Stevens AN, Morris PG, Iles RA, Sheldon PW, Griffiths JR (1984). 5-fluorouracil metabolism monitored invivo by F-19 Nmr. Brit J Cancer.

[33] Deutsch CJ, Taylor JS (1989). New class of 19F pH indicators: fluoroanilines. Biophys J.

[34] Deutsch CJ, Taylor JS (1987). Intracellular pH as measured by 19F NMR. Ann N Y Acad Sci.

[35] Metcalfe JC, Hesketh TR, Smith GA (1985). Free cytosolic Ca-2 + measurements with fluorine labeled indicators using F-19-Nmr. Cell Calcium.

[36] Du WJ, Xu ZQ, Nystrom AM, Zhang K, Leonard JR, Wooley KL (2008). F-19- and fluorescently labeled micelles as nanoscopic assemblies for chemotherapeutic Delivery. Bioconjugate Chem.

[37] Schmieder AH, Caruthers SD, Keupp J, Wickline SA, Lanza GM (2015). Recent advances in (19)Fluorine magnetic resonance imaging with perfluorocarbon emulsions. Engineering (Beijing).

[38] Ashur I, Allouche-Arnon H, Bar-Shir A (2018). Calcium fluoride nanocrystals: tracers for in vivo (19) F magnetic resonance imaging. Angew Chem Int Ed Engl.

[39] Kolouchova K, Sedlacek O, Jirak D, Babuka D, Blahut J, Kotek J, Vit M, Trousil J, Konefal R, Janouskova O, Podhorska B, Slouf M, Hruby M (2018). Self-assembled thermoresponsive polymeric nanogels for (19)F MR imaging. Biomacromolecules.

[40] Sedlacek O, Jirák D, Gálisová A, Jager E, Laaser JE, Lodge TP, Stepanek P, Hrubý M (2018). ^19^F magnetic resonance imaging of injectable polymeric implants with multiresponsive behavior. Chem Mater.

[41] Peng H, Blakey I, Dargaville B, Rasoul F, Whittaker AK (2009). Effect of solvent quality on the solution properties of assemblies of amphiphilic diblock copolymers as potential F-19 MRI agents. Abstr Pap Am Chem.

[42] Tirotta I, Mastropietro A, Cordiglieri C, Gazzera L, Baggi F, Baselli G, Bruzzone MG, Zucca I, Cavallo G, Terraneo G, Baldelli Bombelli F, Metrangolo P, Resnati G (2014). A superfluorinated molecular probe for highly sensitive in vivo(19)F-MRI. J Am Chem Soc.

[43] Gálisova A, Herynek V, Swider E, Sticová E, Pátiková A, Kosinová L, Kříž J, Hájek M, Srinivas M, Jirák D (2018). A trimodal imaging platform for tracking viable transplanted pancreatic islets in vivo: 19F MR, fluorescence and bioluminescence imaging. Mol Imaging Biol.

[44] Srinivas M, Boehm-Sturm P, Figdor CG, de Vries IJ, Hoehn M (2012). Labeling cells for in vivo tracking using (19)F MRI. Biomaterials.

[45] Liang S, Dresselaers T, Louchami K, Zhu C, Liu Y, Himmelreich U (2017). Comparison of different compressed sensing algorithms for low SNR (19) F MRI applications-Imaging of transplanted pancreatic islets and cells labeled with perfluorocarbons. NMR Biomed.

[46] Waiczies H, Guenther M, Skodowski J, Lepore S, Pohlmann A, Niendorf T, Waiczies S (2013). Monitoring dendritic cell migration using 19F/1H magnetic resonance imaging. J Vis Exp.

[47] Weibel S, Basse-Luesebrink TC, Hess M, Hofmann E, Seubert C, Langbein-Laugwitz J, Gentschev I, Sturm VJF, Ye Y, Kampf T, Jakob PM, Szalay AA (2013). Imaging of intratumoral inflammation during oncolytic virotherapy of tumors by F-19-magnetic resonance imaging (MRI). Plos One.

[48] Ahrens ET, Bulte JWM (2013). Tracking immune cells in vivo using magnetic resonance imaging. Nat Rev Immunol.

[49] Stoll G, Basse-Lusebrink T, Weise G, Jakob P (2012). Visualization of inflammation using (19) F-magnetic resonance imaging and perfluorocarbons. Wiley Interdiscip Rev Nanomed Nanobiotechnol.

[50] Nakstad B, Kahler H, Wolfson MR, Lindemann R, Fugelseth D, Shaffer TH, Lyberg T (1999). Perfluorocarbon chemicals do not induce inflammatory responses in human blood leukocytes. Pediatr Res.

[51] Nystrom AM, Bartels JW, Du W, Wooley KL (2009). Perfluorocarbon-loaded shell crosslinked knedel-like nanoparticles: lessons regarding polymer mobility and self-assembly. J Polym Sci Pol Chem.

[52] Cheng C, Powell KT, Khoshdel E, Wooley KL (2007). Polydimethylsiloxane-(PDMS-) grafted fluorocopolymers by a “grafting through” strategy based on atom transfer radical (Co)polymerization. Macromolecules.

[53] Ahrens ET, Zhong J (2013). In vivo MRI cell tracking using perfluorocarbon probes and fluorine-19 detection. NMR Biomed.

[54] Smith GA, Hesketh RT, Metcalfe JC, Feeney J, Morris PG (1983). Intracellular calcium measurements by F-19 Nmr of fluorine-labeled chelators. Proc Natl Acad Sci-Biol.

[55] Robinson SP, Griffiths JR (2004). Current issues in the utility of F-19 nuclear magnetic resonance methodologies for the assessment of tumour hypoxia. Philos T Roy Soc B.

[56] Bar-Shir A, Yadav NN, Gilad AA, van Zijl PCM, McMahon MT, Bulte JWM (2015). Single F-19 probe for simultaneous detection of multiple metal ions using miCEST MRI. J Am Chem Soc.

[57] Srinivas M, Cruz LJ, Bonetto F, Heerschap A, Figdor CG, de Vries IJ (2010). Customizable, multi-functional fluorocarbon nanoparticles for quantitative in vivo imaging using 19F MRI and optical imaging. Biomaterials.

[58] Jacoby C, Temme S, Mayenfels F, Benoit N, Krafft MP, Schubert R, Schrader J, Flogel U (2014). Probing different perfluorocarbons for in vivo inflammation imaging by 19F MRI: image reconstruction, biological half-lives and sensitivity. NMR Biomed.

[59] Krafft MP, Riess JG (2007). Perfluorocarbons: Life sciences and biomedical uses—dedicated to the memory of Professor Guy Ourisson, a true RENAISSANCE man. J Polym Sci Pol Chem.

[60] Mason RP, Antich PP, Babcock EE, Gerberich JL, Nunnally RL (1989). Perfluorocarbon imaging invivo—a F-19 Mri study in tumor-bearing mice. Magn Reson Imaging.

[61] Shimizu M, Kobayashi T, Morimoto H, Matsuura N, Shimano T, Nomura N, Itoh S, Yamazaki M, Iriguchi N, Yamamoto T, Yamai S, Furuta T, Maki T, Mori T (1987). Tumor imaging with anti-Cea antibody labeled F-19 emulsion. Magnet Reson Med.

[62] Zhang C, Moonshi SS, Han YX, Puttick S, Peng H, Magoling BJA, Reid LC, Bernardi S, Searles DJ, Kral P, Whittaker AK (2017). PFPE-based polymeric F-19 MRI agents: a new class of contrast agents with outstanding sensitivity. Macromolecules.

[63] Peng H, Thurecht KJ, Blakey I, Taran E, Whittaker AK (2012). Effect of solvent quality on the solution properties of assemblies of partially fluorinated amphiphilic diblock copolymers. Macromolecules.

[64] Peng H, Blakey I, Dargaville B, Rasoul F, Rose S, Whittaker AK (2009). Synthesis and evaluation of partly fluorinated block copolymers as MRI imaging agents. Biomacromol.

[65] Mao J, Ni PH, Mai YY, Yan DY (2007). Multicompartment micelles from hyperbranched star-block copolymers containing polycations and fluoropolymer segment. Langmuir.

[66] Yusa S, Yamamoto T, Hashidzume A, Morishima Y (2002). Synthesis and characterization of self-associative perfluoroalkyl-end-capped polystyrene. Polym J.

[67] Andruzzi L, Chiellini E, Galli G, Li XF, Kang SH, Ober CK (2002). Engineering low surface energy polymers through molecular design: synthetic routes to fluorinated polystyrene-based block copolymers. J Mater Chem.

[68] Shi ZQ, Holdcroft S (2005). Synthesis and proton conductivity of partially sulfonated poly([vinylidene difluoride-co-hexafluoropropylene]-b-styrene) block copolymers. Macromolecules.

[69] Destarac M, Matyjaszewski K, Silverman E, Ameduri B, Boutevin B (2000). Atom transfer radical polymerization initiated with vinylidene fluoride telomers. Macromolecules.

[70] Lebreton P, Ameduri B, Boutevin B, Corpart JM (2002). Use of original omega-perfluorinated dithioesters for the synthesis of well-controlled polymers by reversible addition-fragmentation chain transfer (RAFT). Macromol Chem Phys.

[71] Monteiro MJ, Adamy MM, Leeuwen BJ, van Herk AM, Destarac M (2005). A “living” radical ab initio emulsion polymerization of styrene using a fluorinated xanthate agent. Macromolecules.

[72] Sawada H (1996). Fluorinated peroxides. Chem Rev.

[73] Sawada H, Ikeno K, Kawase T (2002). Synthesis of amphiphilic fluoroalkoxyl end-capped cooligomers containing oxime-blocked isocyanato segments: architecture and applications of new self-assembled fluorinated molecular aggregates. Macromolecules.

[74] Boutevin B, Diaf KO, Pietrasanta Y, Taha M (1986). Synthesis of Block cotelomers involving a perfluorinated chain and a hydrophilic chain. 1. Use of Fluorinated Telogens with Trichloromethyl End Groups. J Polym Sci Pol Chem.

[75] Su ZH, Wu DC, Hsu SL, McCarthy TJ (1997). Adsorption of end-functionalized poly(ethylene oxide)s to the poly(ethylene oxide)-air interface. Macromolecules.

[76] Kaberov LI, Verbraeken B, Hruby M, Riabtseva A, Kovacik L, Kereiche S, Brus J, Stepanek P, Hoogenboom R, Filippov SK (2017). Novel triphilic block copolymers based on poly(2-methyl-2oxazoline)-block-poly(2-octyl-2-oxazoline) with different terminal perfluoroalkyl fragments: synthesis and self-assembly behaviour. Eur Polym J.

[77] Aasen SN, Pospisilova A, Eichler TW, Panek J, Hruby M, Stepanek P, Spriet E, Jirak D, Skaftnesmo KO, Thorsen F (2015). A novel nanoprobe for multimodal imaging is effectively incorporated into human melanoma metastatic cell lines. Int J Mol Sci.

[78] Langereis S, Keupp J, van Velthoven JLJ, de Roos IHC, Burdinski D, Pikkemaat JA, Grull H (2009). A temperature-sensitive liposomal H-1 CEST and F-19 contrast agent for mr image-guided drug delivery. J Am Chem Soc.

[79] Navath RS, Menjoge AR, Wang B, Romero R, Kannan S, Kannan RM (2010). Amino acid-functionalized dendrimers with heterobifunctional chemoselective peripheral groups for drug delivery applications. Biomacromol.

[80] Thurecht KJ, Blakey I, Peng H, Squires O, Hsu S, Alexander C, Whittaker AK (2010). Functional hyperbranched polymers: toward targeted in vivo F-19 magnetic resonance imaging using designed macromolecules. J Am Chem Soc.

[81] Rabyk M, Galisova A, Jiratova M, Patsula V, Srbova L, Loukotova L, Parnica J, Jirak D, Stepanek P, Hruby M (2018). Mannan-based conjugates as a multimodal imaging platform for lymph nodes. J Mater Chem B.

[82] Hruby M, Filippov SK, Stepanek P (2016). Supramolecular structures and self-association processes in polymer systems. Physiol Res 65 (Supplementum.

[83] Skodova M, Hruby M, Filippov SK, Karlsson G, Mackova H, Spirkova M, Kankova D, Steinhart M, Stepanek P, Ulbrich K (2011). Novel polymeric nanoparticles assembled by metal ion addition. Macromol Chem Phys.

[84] Hruby M, Konak C, Kucka J, Vetrik M, Filippov SK, Vetvicka D, Mackova H, Karlsson G, Edwards K, Rihova B, Ulbrich K (2009). Thermoresponsive, hydrolytically degradable polymer micelles intended for radionuclide delivery. Macromol Biosci.

[85] Dardzinski BJ, Sotak CH (1994). Rapid tissue oxygen tension mapping using 19F inversion-recovery echo-planar imaging of perfluoro-15-crown-5-ether. Magn Reson Med.

[86] Lee H, Price RR, Holburn GE, Partain CL, Adams MD, Cacheris WP (1994). In vivo fluorine-19 MR imaging: relaxation enhancement with Gd-DTPA. J Magn Reson Imaging.

[87] Nam SY, Ricles LM, Suggs LJ, Emelianov SY (2015). Imaging strategies for tissue engineering applications. Tissue Eng Part B-Re.

[88] Chalmers KH, Kenwright AM, Parker D, Blamire AM (2011). (19)F-lanthanide complexes with increased sensitivity for (19)F-MRI: optimization of the MR acquisition. Magnet Reson Med.

[89] Bouchoucha M, van Heeswijk RB, Gossuin Y, Kleitz F, Fortin MA (2017). Fluorinated mesoporous silica nanoparticles for binuclear probes in H-1 and F-19 magnetic resonance imaging. Langmuir.

[90] Longmire M, Choyke PL, Kobayashi H (2008). Clearance properties of nano-sized particles and molecules as imaging agents: considerations and caveats. Nanomedicine (Lond).

[91] Jokerst JV, Lobovkina T, Zare RN, Gambhir SS (2011). Nanoparticle PEGylation for imaging and therapy. Nanomedicine (Lond).

[92] Brown GO, Bergquist C, Ferm P, Wooley KL (2005). Unusual, promoted release of guests from amphiphilic cross-linked polymer networks. J Am Chem Soc.

[93] Du WJ, Nystrom AM, Zhang L, Powell KT, Li YL, Cheng C, Wickline SA, Wooley KL (2008). Amphiphilic hyperbranched fluoropolymers as nanoscopic (19)F magnetic resonance imaging agent assemblies. Biomacromol.

[94] Nurmi L, Peng H, Seppala J, Haddleton DM, Blakey I, Whittaker AK (2010). Synthesis and evaluation of partly fluorinated polyelectrolytes as components in F-19 MRI-detectable nanoparticles. Polym Chem-Uk.

[95] Loukotova L, Kucka J, Rabyk M, Hocherl A, Venclikova K, Janouskova O, Paral P, Kolarova V, Heizer T, Sefc L, Stepanek P, Hruby M (2017). Thermoresponsive beta-glucan-based polymers for bimodal immunoradiotherapy—are they able to promote the immune system?. J Control Release.

[96] Zhang H, Ni PH, He JL, Liu CC (2008). Novel fluoroalkyl end-capped amphiphilic diblock copolymers with pH/temperature response and self-assembly behavior. Langmuir.

[97] Hoffman AS (2008). The origins and evolution of “controlled” drug delivery systems. J Control Release.

[98] Maeda H (2010). Tumor-selective delivery of macromolecular drugs via the EPR effect: background and future prospects. Bioconjug Chem.

[99] Talelli M, Rijcken CJ, van Nostrum CF, Storm G, Hennink WE (2010). Micelles based on HPMA copolymers. Adv Drug Deliv Rev.

[100] Hruby M, Filippov SK, Panek J, Novakova M, Mackova H, Kucka J, Vetvicka D, Ulbrich K (2010). Polyoxazoline thermoresponsive micelles as radionuclide delivery systems. Macromol Biosci.

[101] Jiratova M, Pospisilova A, Rabyk M, Parizek M, Kovar J, Galisova A, Hruby M, Jirak D (2018). Biological characterization of a novel hybrid copolymer carrier system based on glycogen. Drug Deliv Transl Res.

[102] Kucka J, Hruby M, Konak C, Kozempel J, Lebeda O (2006). Astatination of nanoparticles containing silver as possible carriers of 211At. Appl Radiat Isot.

[103] Sedlacek O, Monnery BD, Filippov SK, Hoogenboom R, Hruby M (2012). Poly(2-oxazoline)s–are they more advantageous for biomedical applications than other polymers?. Macromol Rapid Commun.

[104] Fu CK, Herbst S, Zhang C, Whittaker AK (2017). Polymeric F-19 MRI agents responsive to reactive oxygen species. Polym Chem-Uk.

[105] Bogomolova A, Kaberov L, Sedlacek O, Filippov SK, Stepanek P, Kral V, Wang XY, Liu SL, Ye XD, Hruby M (2016). Double stimuli-responsive polymer systems: how to use crosstalk between pH- and thermosensitivity for drug depots. Eur Polym J.

[106] Schmaljohann D (2006). Thermo- and pH-responsive polymers in drug delivery. Adv Drug Deliver Rev.

